# The long non-coding RNA T cell leukemia homeobox 1 neighbor enhances signal transducer and activator of transcription 5A phosphorylation to promote colon cancer cell invasion, migration, and metastasis

**DOI:** 10.1080/21655979.2022.2068781

**Published:** 2022-05-03

**Authors:** Guanyang Chen, Dongbo Lian, Lei Zhao, Zheng Wang, Qiqige Wuyun, Nengwei Zhang

**Affiliations:** a Department of General Surgery, Peking University Ninth School of Clinical Medicine, Beijing, China; b Department of General Surgery, Beijing Shijitan Hospital, Capital Medical University, Beijing, China; c Department of Critical Care Medicine, Beijing Shijitan Hospital, Capital Medical University, Beijing, China

**Keywords:** TLX1NB, STAT5A, colon cancer, invasion, migration, metastasis

## Abstract

Colon cancer is among the most prevalent gastrointestinal tumor types. The long noncoding RNA (lncRNA) T cell leukemia homeobox 1 neighbor (TLX1NB) is up-regulated in colorectal cancer (CRC). However, the functional role of this lncRNA in colon cancer remains unknown. In our study, we investigated the clinical significance of TLX1NB in colon cancer through bioinformatics analysis and explored its role in migration, invasion and metastasis of colon cancer cell with a series of experiments. Firstly, TLX1NB was up-regulated in colon cancer tissues and increased TLX1NB expression was significantly associated with advanced N stages. In wound healing assays and transwell assays, TLX1NB overexpression promoted HCT116 cell migration and invasion while TLX1NB knockdown inhibited SW620 cell migration and invasion. In vivo, TLX1NB knockdown suppressed pulmonary metastasis of SW620 cell and vimentin expression but increased E-cadherin expression. Then, TLX1NB overexpression enhanced signal transducer and activator of transcription 5A (STAT5A) phosphorylation and TLX1NB knockdown suppressed STAT5A phosphorylation. Moreover, the inhibition of STAT5A phosphorylation reversed TLX1NB overexpression-associated increase in HCT116 cell migratory and invasive activity. In conclusion, TLX1NB enhances STAT5A phosphorylation to promote colon cancer cell invasion, migration, and metastasis.

## Graphical abstract

**Figure:**
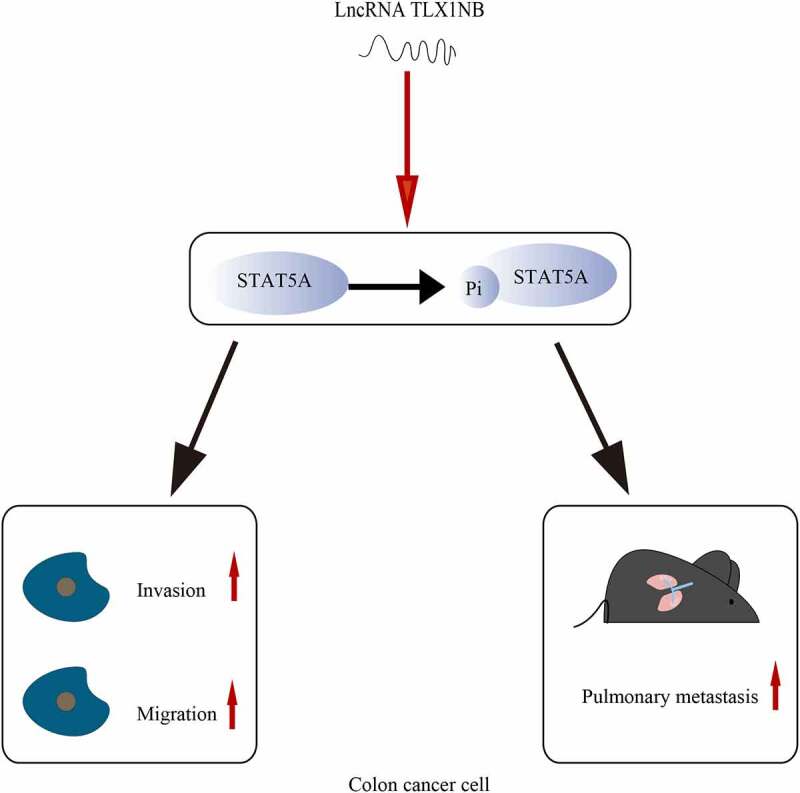

## Highlights

- LncRNA TLX1NB is up-regulated in colon cancer tissues and increased TLX1NB expression is significantly associated with advanced N stages.
- LncRNA TLX1NB promotes the migration, invasion and metastasis of colon cancer cell.
- LncRNA TLX1NB enhances STAT5A phosphorylation in colon cancer cell.
- The inhibition of STAT5A phosphorylation reverses TLX1NB overexpression-associated increase in HCT116 cell migratory and invasive activity.

## Introduction

Colon cancer is among the most prevalent gastrointestinal tumor types, with over 1.14 million new diagnoses and 576,000 deaths globally in 2020 [1]. As one of the major causes of mortality related to colon cancer, metastasis is characterized by the spread of primary tumors to distant sites such as the liver, brain, and lung [2]. Considering the associated high mortality rate, studies are needed to identify reliable therapeutic targets for colon cancer metastasis.

Because they lack the capacity to encode functional proteins, long non-coding RNAs (lncRNAs) were once considered as genetic noise [3–5]. However, recent studies indicated that lncRNAs are involved in the invasion and metastasis of colon cancer [6,7]. The lncRNA T cell leukemia homeobox 1 neighbor (TLX1NB) is encoded in the human 10q24.31 chromosomal region and was reported to be upregulated in colorectal cancer (CRC) tissues [8]. Although elevated TLX1NB levels are correlated with high-grade CRC [8], the functional role of this lncRNA in colon cancer remains unknown.

As a member of the signal transducer and activator of transcription (STAT) family, STAT5A is mostly located in the cytoplasm in an inactivated state [9]. Following stimulation by a series of cytokines, STAT5A is recruited to the Janus kinase/receptor complex and is phosphorylated [10]. STAT5A then translocates to the nucleus where it functions as a transcription factor [11]. Several studies reported that the Janus kinase 2/STAT5 signaling pathway is involved in CRC invasion and metastasis [12,13].

Based on the results of previous studies, we hypothesized that TLX1NB promoted colon cancer invasion and metastasis by enhancing STAT5A phosphorylation. We performed a series of *in vitro* and *in vivo* experiments to investigate the role and mechanism of TLX1NB in colon cancer invasion and metastasis.

## Materials and methods

### Ethics statement

This study was approved by the ethics committee of Beijing Shijitan Hospital (approval no. sjtkyll-lx-2021-61) and was performed in accordance with the Declaration of Helsinki. All animal studies were conducted in accordance with our institutional guidelines for the care and use of laboratory animals.

### Bioinformatics analyses

A colon adenocarcinoma (COAD) dataset consisting of RNA-sequencing and clinical data from 480 COAD tissues and 41 normal colon tissues was downloaded from The Cancer Genome Atlas (TCGA, https://tcga-data.nci.nih.gov/tcga/). These data were analyzed using R 3.6.3 (https://www.r-project.org/). The ggplot2 R package was used to visualize the results. TLX1NB expression was compared between COAD and control tissue samples using the Wilcoxon rank-sum test; and expression levels were further compared among COAD tissues of different N stages using the Kruskal-Wallis and Dunn’s tests. The Survminer R package was used for survival analyses according to the Kaplan-Meier method and log-rank test.

### Cell culture

The HCT116, SW620, and RKO COAD cell lines were obtained from American Type Culture Collection (Manassas, VA, USA). HCT8 cells were obtained from the China Center for Type Culture Collection (Wuhan, China). The NCM460 cell line was purchased from INCELL (San Antonio, TX, USA). The SW620 and HCT116 cell lines were cultured in Dulbecco’s Modified Eagle Medium (Gibco, Grand Island, NY, USA), RKO cells were cultured in Minimum Essential Medium (Gibco), HCT-8 cells were cultured in RPMI-1640 (Gibco), and NCM460 cells were cultured in M300F (INCELL). All cell culture media were supplemented with 10% fetal bovine serum (Gibco) and penicillin/streptomycin (Hyclone, Logan, UT, USA), and the cells were cultured in a humidified incubator at 37°C and 5% CO_2_.

The STAT5 inhibitor pimozide was obtained from MedChemExpress (Monmouth Junction, NJ, USA) and used to treat the colon cancer cells at a dose of 10 μM for 24 h.

### Lentivirus infection

For TLX1NB knockdown, two DNA oligonucleotides encoding TLX1NB-specific short hairpin RNA (shRNA) sequences (target sequences: shTLX1NB-1, 5′-GTCAAGAAAGCAGTGTTTGCGT-3′; shTLX1NB-2, 5′-AATGTTTCTTCCTTATCTGAG-3′) were inserted into the pLVX-shRNA2-puro lentiviral vectors. To overexpress TLX1NB, the human TLX1NB cDNA sequence (synthesized by Tsingke, Beijing, China) was inserted into the pLVX-IRES-ZsGreen1 lentiviral vector using the following primers: TLX1NB forward, 5′-GGATCTATTTCCGGTGAATTCGCCAGAGATAGAGGGACGGAG-3′; TLX1NB reverse, 5′-CGCTCTAGAACTAGTCTCGAGTTTTTTTTTTTTTTTAAAGGTTTTCTATACA-3′. Lentiviral particles were produced by co-transfecting these vectors and the corresponding packaging plasmids into 293 T cells using Lipofectamine 2000 (Thermo Fisher Scientific, Waltham, MA, USA). At 48 h post-transfection, the lentivirus-containing supernatants were collected. The SW620 colon cancer cells were then infected with prepared pLVX-shTLX1NB-1-puro (LV-shTLX1NB-1) or pLVX-shTLX1NB-2-puro (LV-shTLX1NB-2) particles; the pLVX-shRNA2-puro vector was used as a negative control (LV-shNC). HCT116 cells were infected with pLVX-IRES-ZsGreen1-TLX1NB (LV-oeTLX1NB) or empty vector control (pLVX-IRES-ZsGreen1). After appropriate lentiviral infection and selection, TLX1NB expression in these cells was assessed using real-time quantitative polymerase chain reaction (RT-qPCR).

### RT-qPCR

RT-qPCR was performed as previously described [14]. TRIzol (Thermo Fisher Scientific) was used to extract cellular RNA, after which an All-in-One First-Strand cDNA Synthesis Kit (GeneCopoeia, Rockville, MD, USA) was used to prepare cDNA. All qPCR steps were conducted on an QuantStudio™ 6 Flex Real-Time PCR System (Applied Biosystems, Foster City, CA, USA) using iQ SYBR Green Supermix (Bio-Rad, Hercules, CA, USA). The year of manufacture of QuantStudio™ 6 Flex Real-Time PCR System is 2015. The primers used for these analyses were as follows: TLX1NB forward, 5′-GACGAGGCAGGAGCAGAGAG-3′; TLX1NB reverse, 5′-CTTGAGTGGGGATAGATGGAGA-3′; GAPDH forward, 5′-ACAGCCTCAAGATCATCAGC-3′; GAPDH reverse, 5′-GGTCATGAGTCCTTCCACGAT-3′. The 2^−ΔΔCt^ method was used to assess relative gene expression [15], with GAPDH used as the normalization control.

### Western blotting

Western blotting was performed as previously described [16]. Lysis buffer (Beyotime, Beijing, China) was used to extract total protein from the cells, and protein levels were quantified using the BCA assay (Beyotime). The proteins were then separated using 10% sodium dodecyl sulfate-polyacrylamide gel electrophoresis, transferred onto polyvinylidene fluoride membranes, and blocked at room temperature for 1 h using 5% nonfat milk. The membranes were incubated overnight with antibodies specific for STAT5A (1:1000, 13179-1-AP, Proteintech, Rosemont, IL, USA), p-STAT5A (1:2000, 80115-1-RR, Proteintech), or GAPDH (1:6000, 10494-1-AP, Proteintech) at 4°C. After three washes with Tris-buffered saline containing Tween-20, the membranes were incubated for 2 h with a secondary antibody (1:3000, 14708 Cell Signaling Technology, Danvers, MA, USA) at room temperature, after which an Odyssey® CLx imaging system (LI-COR Biosciences, Lincoln, NE, USA) was used to visualize the protein bands. The year of manufacture of Odyssey® CLx imaging system is 2015. GAPDH served as a loading control to normalize the band densities.

### Immunohistochemistry

Lung tissues were fixed using 10% formaldehyde, embedded in paraffin, and cut into 5 μm sections. These sections were deparaffinized and rehydrated according to previously described methods [17], incubated for 10 min in 3% H_2_O_2_, and washed using phosphate-buffered saline (PBS). After blocking with 3% bovine serum albumin, the sections were incubated overnight with antibodies specific for E-cadherin (1:1000, 20874-1-AP, Proteintech) and anti-vimentin (1:5000, 10366-1-AP, Proteintech) at 4°C. The sections were then incubated with the appropriate secondary antibodies, stained with diaminobenzidine, and counterstained with hematoxylin as described previously [18]. The slides were dehydrated and sealed with neutral gum.

### Wound healing assay

The cells were added to 6-well plates and grown until ~90% confluence, and then a pipette tip was used to generate a scratch wound on the monolayer surface. After removing detached cells by rinsing with PBS, the cells were cultured in a serum-free medium. Cell migration on the plate was measured at 0 and 24 h post-wounding under an ECLIPSE TS100 inverted microscope (605870, Nikon, Tokyo, Japan). ImageJ software (NIH, Bethesda, MD, USA) was used to measure wound closure as follows: wound closure (%) = [(A_t=0h_ − A_t=Δh_)/A_t=0h_] × 100% [19], where A_t=0h_ and A_t=Δh_ were the wound areas at 0 h and time Δ h, respectively.

### Transwell assay

A Transwell Chamber 3422 (Corning, Inc., Corning, NY, USA) was used to assess the migratory and invasive activity of cancer cells. The chambers were coated with Matrigel solution (BD Biosciences, Franklin Lakes, NJ, USA) for invasion assays. Appropriately transduced cells were cultured for 24 h in serum-free medium, after which the upper chamber of a transwell insert was loaded with 200 μL of the cell suspensions (4 × 10^4^ cells) and the lower chamber of a 24-well plate was loaded with complete medium supplemented with 10% fetal bovine serum. Following 48 h of incubation, cells remaining in the upper chamber were removed with a cotton swab, whereas the remaining migratory and invasive cells were fixed using 4% paraformaldehyde, stained with crystal violet, and counted in three independent fields of view via an ECLIPSE TS100 inverted microscope (Nikon).

### In vivo metastasis assay

A model of pulmonary metastasis was established using BALB/c nude mice (4–6 weeks old) purchased from the China Scientific Research Institute of Animals (Beijing, China). Twelve nude mice were randomly divided into two groups. 0.1 mL of PBS containing 2 × 10^6^ SW620 cells that had been transduced with LV-shTLX1NB-1 or LV-shNC was injected into each animal via the tail vein. On day 50 post-injection, the animals were euthanized, and the number of metastatic nodes visible in the lungs of the mice was counted. Lung tissue was harvested for western blotting, qPCR, and immunohistochemistry staining.

### Statistical analyses

Data from the *in vitro* and *in vivo* experiments were analyzed using GraphPad Prism 7 software (GraphPad, Inc., La Jolla, CA, USA) and were compared using two-tailed Student’s *t*-tests or one-way analysis of variance with Tukey’s post hoc test as appropriate. The threshold for significance was set at P < 0.05.

## Results

We investigated the role of TLX1NB in colon cancer invasion and metastasis. First, we scanned TCGA and identified TLX1NB as lncRNA that is upregulated in colon cancer. We confirmed that TLX1NB promoted the migration and invasion of colon cancer cells *in vitro* and enhanced the metastasis of colon cancer cells *in vivo*. In addition, we found that TLX1NB promoted the invasion and metastasis of colon cancer cells by enhancing STAT5A phosphorylation. These data suggest that TLX1NB can be used as a target for the diagnosis and treatment of colon cancer.

### Upregulation of TLX1NB in COAD

**Figure 1.**
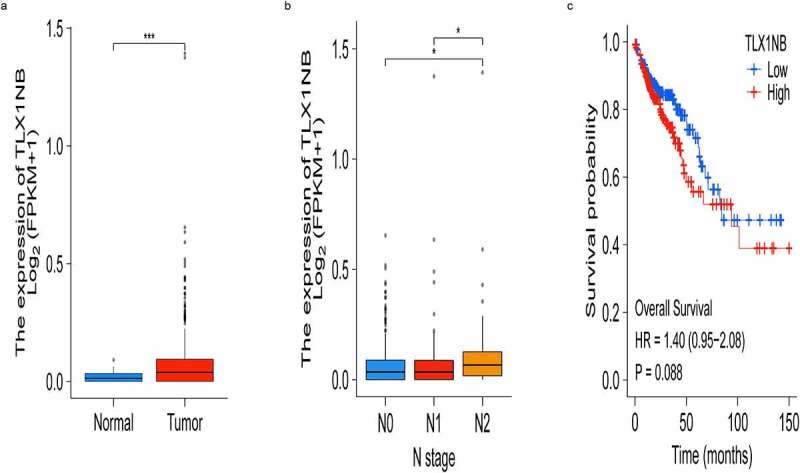
The expression of TLX1NB in COAD and normal colon tissues based on TCGA. (a) The expression levels of TLX1NB in COAD and normal colon tissues. (b) The expression levels of TLX1NB in N0, N1 and N2 COAD. (c) Survival analysis of COAD patients with low and high TLX1NB expression level. *P < 0.05; ***P < 0.001.

We firstly evaluated TLX1NB expression levels in COAD tissues and control samples from TCGA database. We found that TLX1NB expression was significantly upregulated in COAD tissues (P < 0.001; Figure 1(a)). Using the American Joint Committee on Cancer staging system, we found that TLX1NB was expressed at higher levels in N2 stage COAD samples than in N0 (P < 0.05) or N1 (P < 0.05) stage samples (Figure 1(b)). Survival analyses of this patient cohort revealed that higher levels of TLX1NB were associated with worse survival outcomes compared to those of patients expressing low levels of this lncRNA, although the difference was not significant (P = 0.088; Figure 1(c)).

### Analysis of TLX1NB expression in colon cancer cell lines

**Figure 2.**
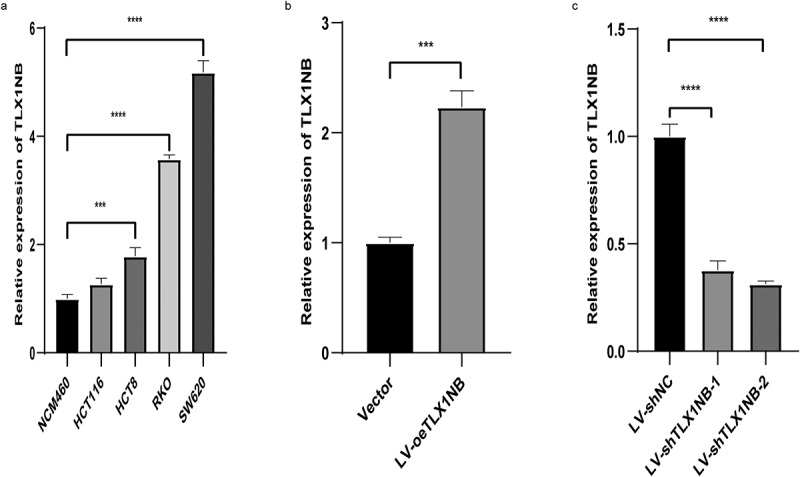
Analysis of TLX1NB expression levels by qPCR in normal colonic epithelial cells and different colon cancer cells. (a) The expression levels of TLX1NB in NCM460, SW620, RKO, HCT8 and HCT116 cell lines. (b) LV-oeTLX1NB transfection enhanced the expression of TLX1NB in HCT116 cells. (c) LV-shTLX1NB-1 and LV-shTLX1NB-2 transfection inhibited the expression of TLX1NB in SW620 cells. ***P < 0.001; ****P < 0.0001.

To expand on the above results, TLX1NB expression levels were assessed using qPCR in the HCT116, HCT8, RKO, NCM460, and SW620 cell lines. Relative to that in NCM460 cells, TLX1NB was significantly upregulated in RKO, SW620, and HCT8 cells (all P < 0.001, Figure 2(a)). We then selected SW620 and HCT116 cells, which exhibited high and low levels of TLX1NB expression, respectively, for subsequent analysis. HCT116 cells were used to generate a colon cancer cell line stably overexpressing TLX1NB via transduction of the LV-oeTLX1NB vector, leading to pronounced TLX1NB upregulation (P < 0.001, Figure 2(b)). In contrast, TLX1NB was stably knocked down in SW620 cells; both the LV-shTLX1NB-1 and LV-shTLX1NB-2 vectors achieved significant TLX1NB knockdown (both P < 0.0001, Figure 2(c)).

## *TLX1NB promoted* in vitro *colon cancer cell migration in wound healing assays*

**Figure 3.**
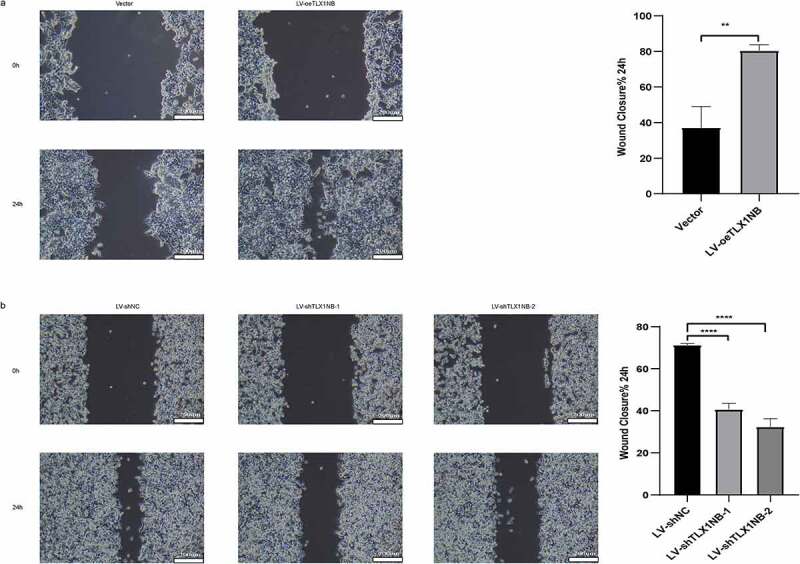
The effects of TLX1NB on wound healing of colon cancer cells in vitro. (a) Wound healing assays in HCT116 cells transfected with LV-oeTLX1NB or empty vector control. (b) Wound healing assays in SW620 cells transfected with LV-shTLX1NB-1, LV-shTLX1NB-2 or LV-shNC. **P < 0.01; ****P < 0.0001.

In wound healing assays, overexpression of TLX1NB was found to enhance the migration of HCT116 cells, as determined based on the percentage wound closure (P < 0.01, Figure 3(a)), whereas TLX1NB knockdown in SW620 cells had the opposite effect (both P < 0.0001, Figure 3(b)).

## *TLX1NB enhanced* in vitro *colon cancer cell migration and invasion in transwell assays*

**Figure 4.**
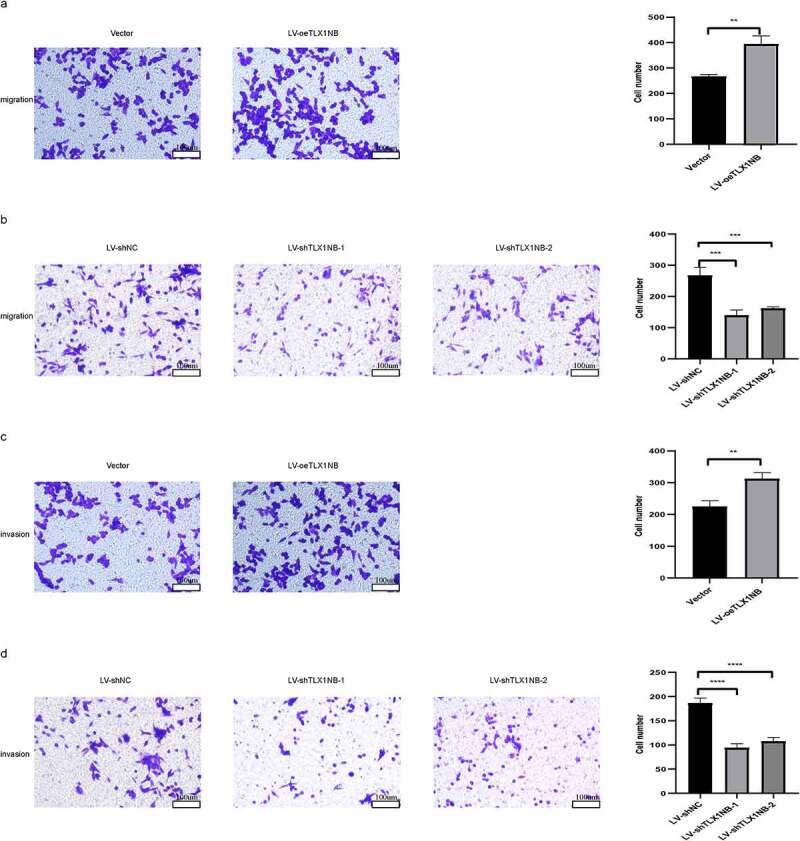
The effects of TLX1NB on migration and invasion of cancer cells in vitro. (a) Transwell migration assays in HCT116 cells transfected with LV-oeTLX1NB or empty vector control. (b) Transwell migration assays in SW620 cells transfected with LV-shTLX1NB-1, LV-shTLX1NB-2 or LV-shNC. (c) Transwell invasion assays in HCT116 cells transfected with LV-oeTLX1NB or empty vector control. (d) Transwell invasion assays with in SW620 cells transfected with LV-shTLX1NB-1, LV-shTLX1NB-2 or LV-shNC. **P < 0.01; ***P < 0.001; ****P < 0.0001.

Next, we assessed the impact of TLX1NB expression on the migratory and invasive activity of colon cancer cells using a transwell assay system. The results showed that TLX1NB overexpression significantly enhanced HCT116 cell migration (P < 0.01; Figure 4(a)), whereas TLX1NB knockdown inhibited SW620 cell migration (both P < 0.001; Figure 4(b)). Similarly, TLX1NB overexpression increased the invasive activity of HCT116 cells (P < 0.01; Figure 4(c)), whereas TLX1NB knockdown suppressed this activity of SW620 cells (both P < 0.0001; Figure 4(d)).

## *TLX1NB knockdown inhibited* in vivo *SW620 cells pulmonary metastasis*

**Figure 5.**
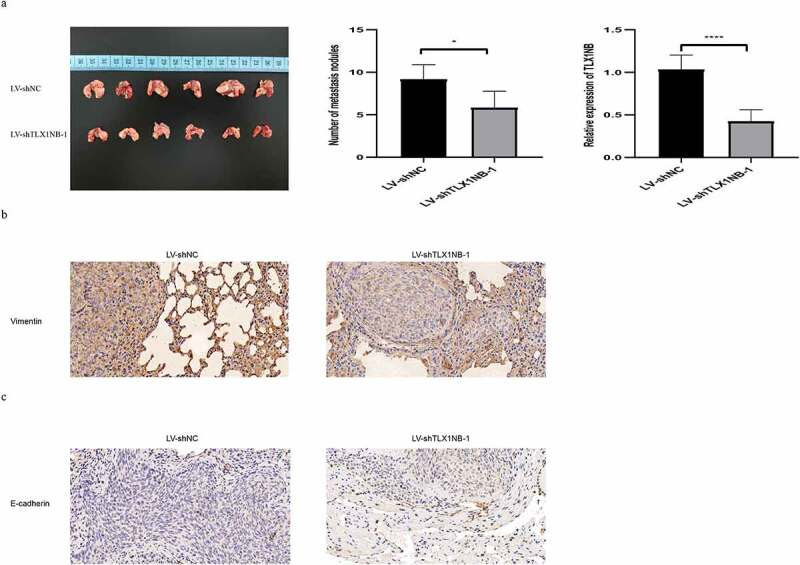
Knockdown of TLX1NB suppressed pulmonary metastasis of SW620 cells in vivo. (a) Images of representative mouse lungs in LV-shNC or LV-shTLX1NB-1 group. (b) The numbers of metastasis tumor nodules in LV-shNC or LV-shTLX1NB-1 group. (c) The qPCR results of TLX1NB in metastasis tumor nodules of LV-shNC or LV-shTLX1NB-1 group. (d) Representative IHC images of Vimentin staining in metastasis tumor nodules of LV-shNC or LV-shTLX1NB-1 group. (e) Representative IHC images of E-Cadherin staining in metastasis tumor nodules of LV-shNC or LV-shTLX1NB-1 group. *P < 0.05; ****P < 0.0001.

We developed an *in vivo* model of pulmonary metastasis of colon cancer in nude mice. There were significantly fewer metastatic lung nodules in mice injected with SW620 cells in which TLX1NB had been knocked down (LV-shTLX1NB-1) than in those injected with LV-shNC-transduced cells (P < 0.01; Figure 5(a,b)). TLX1NB expression levels were significantly lower in pulmonary metastases from mice in the LV-shTLX1NB-1 group than in those in the LV-shNC group (P < 0.0001; Figure 5(c)). Immunohistochemical staining of these tumor samples further revealed decreased vimentin levels in metastatic pulmonary nodes in the LV-shTLX1NB-1 group relative to in the LV-shNC group (P < 0.05; Figure 5(d)), whereas E-cadherin levels were elevated in pulmonary metastases from the LV-shTLX1NB-1 group compared to those in the LV-shNC group (P < 0.05; Figure 5(e)).

### TLX1NB promoted STAT5A phosphorylation

**Figure 6.**
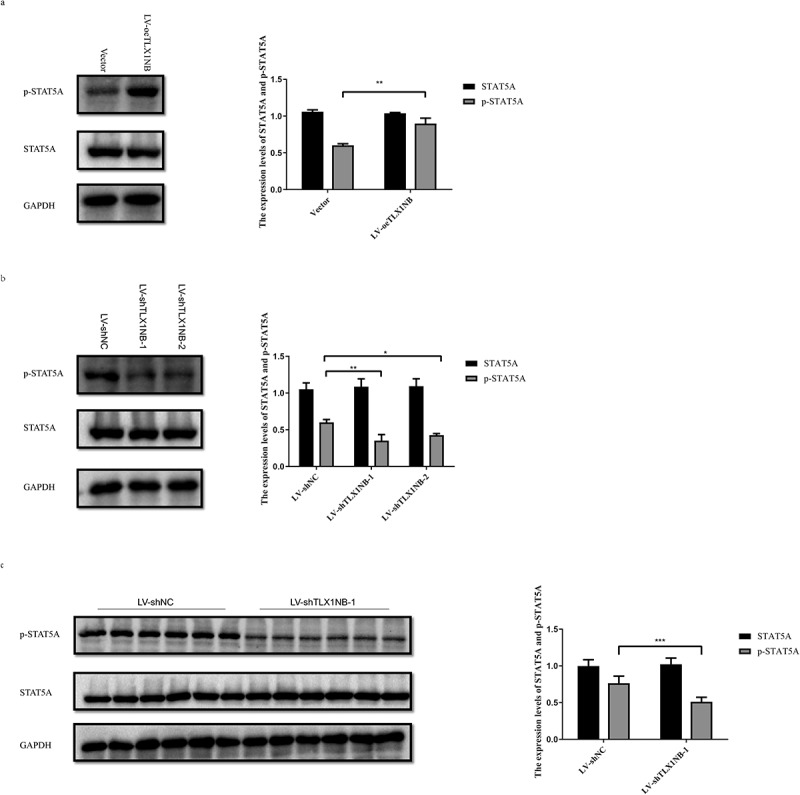
TLX1NB enhanced the phosphorylation of STAT5A. (a) The western blotting results of STAT5A and p-STAT5A in HCT116 cells transfected with LV-oeTLX1NB or empty vector control in vitro. (b) The western blotting results of STAT5A and p-STAT5A in SW620 cells transfected with LV-shTLX1NB-1, LV-shTLX1NB-2 or LV-shNC in vitro. (c) The western blotting results of STAT5A and p-STAT5A in metastasis tumor nodules of LV-shNC or LV-shTLX1NB-1 group. *P < 0.05; **P < 0.01; ***P < 0.001.

We then examined the total and phosphorylated STAT5A levels following knockdown or overexpression of TLX1NB *in vitro*. When TLX1NB was overexpressed, HCT116 cells exhibited increased p-STAT5A levels (P < 0.01) without a concomitant increase in total STAT5A expression levels (Figure 6(a)), whereas TLX1NB knockdown in SW620 cells was associated with decreased p-STAT5A levels (both P < 0.05) without a change in total STAT5A expression levels (Figure 6(b)). Similarly, in our pulmonary metastasis model system, p-STAT5A levels were lower in the metastatic nodules from mice in the LV-shTLX1NB-1 group than in those in the LV-shNC group (P < 0.001); and the total expression levels of STAT5A were unchanged (Figure 6(c)).

### Inhibition of STAT5A phosphorylation reversed TLX1NB overexpression-induced enhancement in HCT116 cell migration and invasion

**Figure 7.**
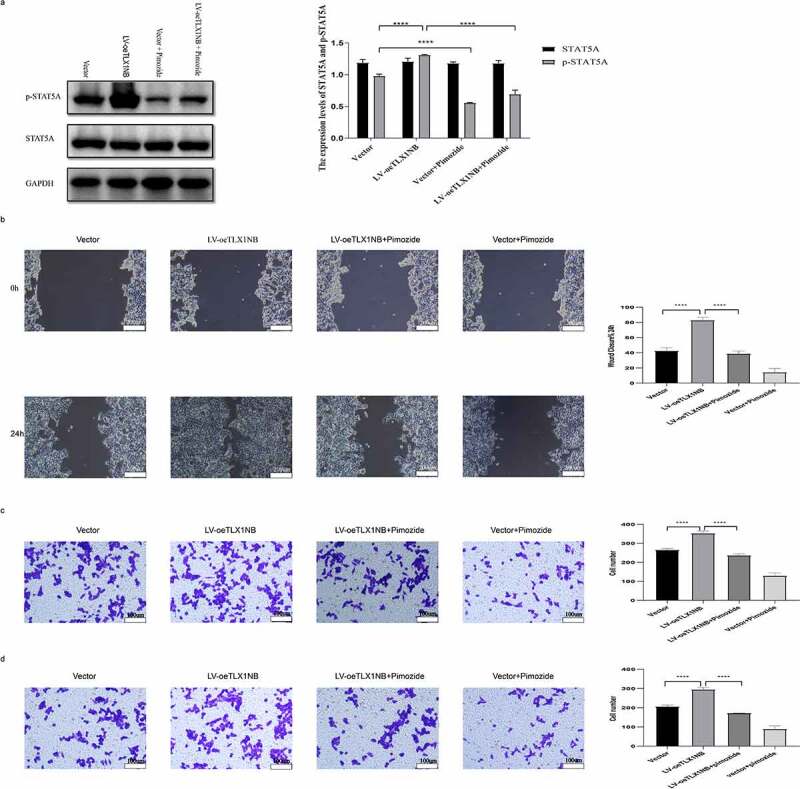
The inhibition of STAT5A phosphorylation reversed TLX1NB overexpression-associated increases in HCT116 cell migratory and invasive activity. Notes: TLX1NB-overexpressing HCT116 cells and their controls were treated in the absence or presence of pimozide (10 μM) for 24 hours. (a) The STAT5A and p-STAT5A protein expression levels in TLX1NB-overexpressed HCT116 cells and their negative controls with or without pimozide treatment. (b) Wound healing assays for TLX1NB-overexpressed HCT116 cells and their negative controls with or without pimozide treatment. (c) Transwell migration assays for TLX1NB-overexpressed HCT116 cells and their negative controls with or without pimozide treatment. (d) Transwell invasion assays for TLX1NB-overexpressed HCT116 cells and their negative controls with or without pimozide treatment. ****P < 0.0001.

Treatment with pimozide, a specific STAT5 inhibitor, markedly reduced p-STAT5A levels in TLX1NB-overexpressing HCT116 cells (P < 0.0001) without reducing total STAT5A expression levels (Figure 7(a)). In wound healing assays, pimozide treatment markedly ablated the enhanced migration of HCT116 cells in which TLX1NB was overexpressed (P < 0.0001; Figure 7(b)). Transwell migration and invasion assays showed that pimozide treatment of HCT116 cells reversed TLX1NB overexpression-associated enhancement of migration and invasion (both P < 0.0001; Figure 7(c,d)).

## Discussion

Colon cancer is one of the most common human malignancies [20,21]. LncRNAs have been reported to play vital roles in regulating the invasion, migration, and metastasis of colon cancer cells [22–24]. For example, overexpression of LINC00662 increases colon cancer cell migration and invasion [22], whereas upregulation of LNC01082 inhibits SW480 and SW620 cell migration and invasion [23]. Knockdown of ENST00000455974 inhibits the migration of colon cancer cells [24]. TLX1NB was recently reported to be upregulated in CRC tissues relative to that in adjacent normal tissues [8]. In addition, elevated TLX1NB levels were correlated with higher-grade CRC [8]. However, the specific mechanistic role of TLX1NB in colon cancer remains unclear.

We initially explored the clinical relevance of TLX1NB in colon cancer through a series of bioinformatic analyses. This approach revealed TLX1NB upregulation in COAD tissues relative to normal tissues. This upregulation is associated with more advanced N stages in patients with COAD, suggesting a potential link between this lncRNA and lymph node metastasis. Lymph node metastasis is one of the main mechanisms of colon cancer metastasis and directly affects patient prognosis [25]. Then, we found that *in vitro*, TLX1NB overexpression enhanced HCT116 cell migration and invasion, whereas its knockdown suppressed these effects in SW620 cells. In a model of pulmonary metastasis, TLX1NB knockdown inhibited SW620 cell metastasis to the lungs and reduced vimentin expression in the resulting metastatic lesions, whereas E-cadherin levels were increased. E-cadherin and vimentin are closely associated with epithelial-to-mesenchymal transition, an important process in colon cancer invasion and metastasis characterized by downregulation of epithelial genes, including E-cadherin and zona occludens 1, and upregulation of mesenchymal markers, including N-cadherin and vimentin [26–29]. These data indicate a link between the expression of TLX1NB and induction of epithelial-to-mesenchymal transition in colon cancer, with this lncRNA acting in an oncogenic manner to drive migration, invasion, and metastasis.

To further investigate the mechanisms governing TLX1NB-mediated regulation of colon cancer cell invasion and metastasis, we detected the expression levels of STAT5A and p-STAT5A following TLX1NB knockdown or overexpression. Although TLX1NB overexpression resulted in enhanced STAT5A phosphorylation, its knockdown had the opposite effect, whereas there were no changes in total STAT5A levels *in vitro*. Similar changes were observed in the pulmonary metastasis model after knockdown of TLX1NB *in vivo*. Thus, TLX1NB may drive colon cancer cell migration and invasion, at least in part by promoting STAT5A phosphorylation. To test this possibility directly, we used the STAT5 inhibitor pimozide. Pimozide, which is clinically used to treat schizophrenia, has been reported to inhibit STAT5 phosphorylation in chronic myelogenous leukemia, T-cell prolymphocytic leukemia, osteosarcoma, and breast cancer [10,30–33]. In rescue assays in which HCT116 cells were treated with pimozide, inhibition of STAT5A phosphorylation reversed the enhanced migration and invasion of colon cancer cells following TLX1NB overexpression, suggesting that pimozide has clinical value for treating colon cancer. STAT5A has been reported to drive oncogenesis in many malignancies including lung cancer, prostate cancer, gastric cancer, and ductal carcinoma *in situ* [34–37]. Non-phosphorylated STAT5A has also been reported to suppress human and murine colon cancer cell growth *in vivo*, which is consistent with our data [38].

In this study, we investigated the role of TLX1NB in colon cancer invasion and metastasis through a series of wet and dry experiments and reported that TLX1NB enhanced STAT5A phosphorylation to promote colon cancer cell invasion, migration, and metastasis for the first time. However, this study had some limitations. We did not compare TLX1NB expression levels in clinical colon cancer samples using qPCR, nor did we clarify the mechanism by which this lncRNA promotes STAT5A phosphorylation. Therefore, additional studies are needed to evaluate these factors.

## Conclusion

Together, these results show that the lncRNA TLX1NB promotes colon cancer cell migratory, invasive, and metastatic activity, at least in part by inducing robust STAT5A phosphorylation. Thus, the TLX1NB/STAT5A axis may represent a valuable therapeutic target in patients affected by this malignancy.

## Data Availability

The data that support the findings of this study are available from the corresponding author upon reasonable request (https://doi.org/10.6084/m9.figshare.19642812.v1).
